# A computational model of rabbit geometry and ECG: Optimizing ventricular activation sequence and APD distribution

**DOI:** 10.1371/journal.pone.0270559

**Published:** 2022-06-30

**Authors:** Robin Moss, Eike M. Wülfers, Raphaela Lewetag, Tibor Hornyik, Stefanie Perez-Feliz, Tim Strohbach, Marius Menza, Axel Krafft, Katja E. Odening, Gunnar Seemann

**Affiliations:** 1 Institute for Experimental Cardiovascular Medicine, University Heart Center Freiburg ⋅ Bad Krozingen, Medical Center—University of Freiburg, Freiburg, Germany; 2 Faculty of Medicine, University of Freiburg, Freiburg, Germany; 3 Department of Radiology, Medical Physics, University Medical Center Freiburg, Freiburg, Germany; 4 Department of Cardiology and Angiology I, Heart Center University of Freiburg, Medical Faculty, Freiburg, Germany; 5 Translational Cardiology, Department of Cardiology and Department of Physiology, University Hospital Bern, Bern, Switzerland; University of Minnesota, UNITED STATES

## Abstract

Computational modeling of electrophysiological properties of the rabbit heart is a commonly used way to enhance and/or complement findings from classic lab work on single cell or tissue levels. Yet, thus far, there was no possibility to extend the scope to include the resulting body surface potentials as a way of validation or to investigate the effect of certain pathologies. Based on CT imaging, we developed the first openly available computational geometrical model not only of the whole heart but also the complete torso of the rabbit. Additionally, we fabricated a 32-lead ECG-vest to record body surface potential signals of the aforementioned rabbit. Based on the developed geometrical model and the measured signals, we then optimized the activation sequence of the ventricles, recreating the functionality of the Purkinje network, and we investigated different apico-basal and transmural gradients in action potential duration. Optimization of the activation sequence resulted in an average root mean square error between measured and simulated signal of 0.074 mV/ms for all leads. The best-fit T-Wave, compared to measured data (0.038 mV/ms), resulted from incorporating an action potential duration gradient from base to apex with a respective shortening of 20 ms and a transmural gradient with a shortening of 15 ms from endocardium to epicardium. By making our model and measured data openly available, we hope to give other researchers the opportunity to verify their research, as well as to create the possibility to investigate the impact of electrophysiological alterations on body surface signals for translational research.

## Introduction

The heart is a fascinatingly complex organ, whose electrophysiological properties have been the subject of many studies. Yet, a vast amount of its characteristics are still to be uncovered by scientific research. In clinical and experimental laboratories, the rabbit model has shown to be a good compromise between mouse and larger (farm animal) models, being more similar to the human in terms of electrophysiological properties (see for example the work by Nerbonne [1]), and also cheaper and easier in handling. Over time, computational modeling has shown its potential to not just aid, but also to allow applications outside the scope of classic wet experimental work. One of these applications is the possibility to investigate the impact of local electrophysiological properties within the heart on signals measured on the torso [2–4]. However, to do so, one needs not only a geometrical model of the heart but of the full inhomogeneous torso itself and so far such models, to our knowledge, do not openly exist for the rabbit.

Prior, anatomical modelling has mainly focused on the rabbit ventricles. The most commonly used model is probably the “San Diego” rabbit by Vetter & McCulloch [5]. For that model, a rabbit heart was fixed and reconstructed slice by slice. Another published ventricular model is by Bishop *et al*. [6], where a fixed heart was imaged using a high-resolution MRI. In both previous models, the local myocyte orientation was calculated based on image information. Most recently, Krishnamoorthi *et al*. [7] used a similar approach using data from diffusion tensor MRI to obtain the myocyte orientation. The only model including the atria thus far was published by Stephenson *et al*. [8]. Here, multiple fixed rabbit and rat hearts were imaged using contrast enhanced micro-CT, with focus on the cardiac conduction system. A more detailed description of the models and their respective usages across different studies can be found in Arevalo *et al*. [9]. These four models have a clear advantage in terms of the underlying image resolution, as well as information obtained about myocyte orientation compared to the approach presented within this manuscript. But none of these could validate their electrophysiological parameterization using measured body surface signals, and the impact that heart extraction as well as fixation have on the heart itself is not known—a gap we try to bridge within this study. Similar to what we propose within this work, the group by Bin He [10, 11] have used CT imaging to create a ventricular model coupled with recorded surface signals, but use it to ‘image’ the activation sequence of a heart which was paced from the left ventricle *in vivo*. As their focus was on recreating the local activation times in the ventricles, their model does not include any information about the underlying repolarization dynamics and only minimal information regarding atria and other organs in the torso. Lastly, as the ventricles were paced from one side the recorded BSPM does not contain any information about the normal physiological excitation pattern *via* the Purkinje system.

Consequently, within this work, in order to strengthen the bridge between traditional lab work and computational modeling, we present the first openly available geometrical model of the rabbit heart and torso, developed to be used in computational modeling. Further, in order to parameterize and validate modeled electrophysiological properties, we designed and manufactured a 32-lead ECG-vest, enabling us to record a body surface potential map (BSPM) from known torso locations. Using the geometrical model and the QRS part of the measured signals, we then initially optimize a ventricular activation sequence (*i.e*. depolarization patterns) to recreate the functionality of the Purkinje network. Subsequently, using the T-Wave part of measured signals, we investigate potential physiological apico-basal as well as transmural action potential duration (APD) gradients (*i.e*. repolarization patterns) within the ventricles as values described in literature contradict one another [12–14].

## Materials and methods

### Online availability of materials

All used data are made available online via Zenodo:

Link: https://zenodo.org/record/6340066 DOI:10.5281/zenodo.6340066.

This includes:

- CT image data of heart and torso.
- Meshed geometries of heart (including myocyte orientation) and torso in VTK file format.
- Position of electrodes.
- Recorded unfiltered BSPM recordings.
- Filtered and averaged signals.
- Optimized Purkinje tree and resulting activation sequence.
- Segmented endocardial surface including papillary muscles.

### CT recordings

The CT recordings were acquired using a Toshiba Aquilion ONE CT-Scanner at the radiology department of the Uniklinik Freiburg. To enhance the contrast between blood and tissue, contrast agent (Imeron 300M by Bracco) was injected with a dosage of approximately 2 mL/kg (based on guidelines for infants) at a flow of 0.2 mL/s (lowest possible rate). The resulting image resolution was 0.351 mm * 0.351 mm * 0.5 mm, with an acquisition window of 18 cm * 18 cm * 32 cm. During the recording, the rabbit (wildtype New Zealand white, female, 6 months, 3.0 kg) was anesthestized with S-ketamine and xylazine (12.5/3.75 mg/kg intramuscularly (IM), followed by 1–2.5 mL/kg/h intravenously (IV) to maintain anesthesia during the duration of CT). This anesthesia protocol was chosen as it does not appear to have a significant impact on cardiac repolarization, see Odening *et al*. [15]. All animal care and experimental procedures were performed in compliance with EU legislation (directive 2010/63/EU) and the German (TierSchG and TierSchVersV) animal welfare law, after approval by the local Institutional Animal Care and Use Committees in Germany (Regierungspraesidium Freiburg; approval number G18/118). Animal housing and handling was in accordance with good animal practice as defined by the Federation of European Laboratory Animal Science Association, FELASA.

### BSPM recordings

**Fig 1:**
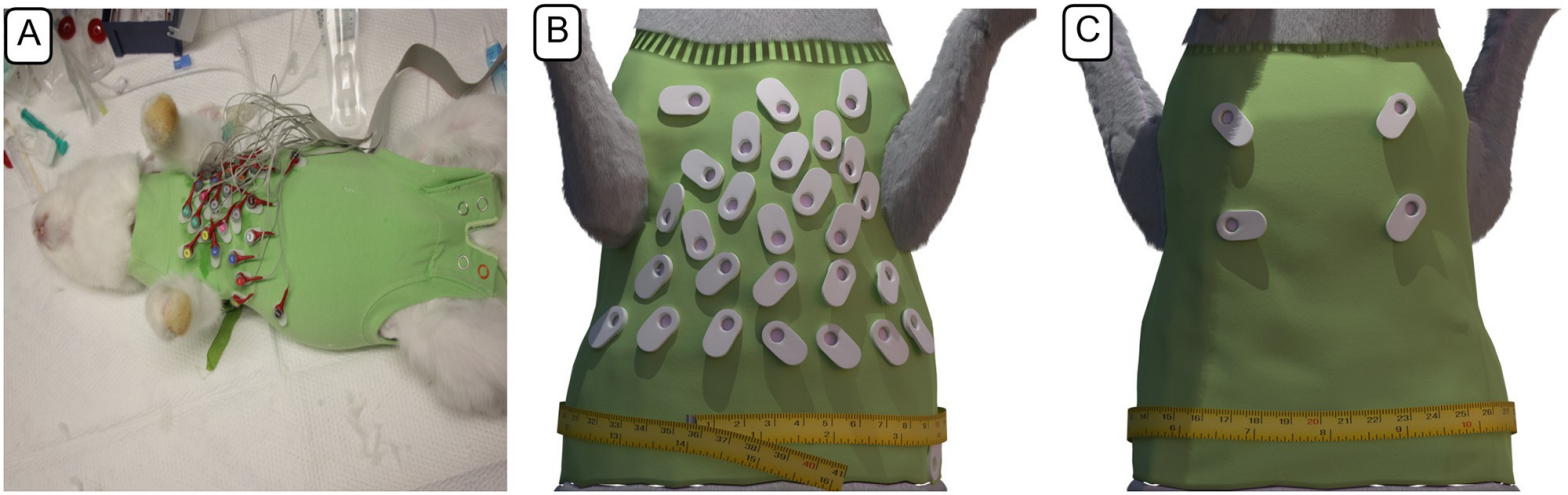
Rabbit 32-lead ECG-vest. A: 32-lead ECG-vest designed to record body surface potentials on the rabbit torso in practical use; B: Rendered illustration of the vest from the anterior side; C: posterior view.

Body surface potentials were recorded using a self-fabricated vest in combination with a 32-channel EEG active-electrode ActiveTwo system (BioSemi B.V., Amsterdam, the Netherlands) with a sampling frequency of 2048Hz. Prior to the recording, the rabbit was anaesthetised as described in the section before. The rabbit was then shaved on the anterior surface of the chest and on its back—corresponding to the regions where the electrodes were placed later on. Electrode gel was used to reduce impedance between skin and electrodes. The positions of electrodes were selected based on the originating field distribution in a test simulation using the ‘SanDiego’ ventricular model [5] and a rough torso model (without any further organs), based on prior MRI recordings from Odening *et al*. [16]. The placement and respective order of signals are shown in Fig 1. The exact location of electrodes was extracted from the CT imaging data, as the rabbit was wearing the vest during the CT measurement (without electrodes in the sockets). Unfortunately, BSPM recordings done immediately prior to the CT imaging had to be discarded as later analyses showed an irregular and high breathing rate (> 1 Hz), which is an indicator that the rabbit had potentially not yet reached the desired depth of anesthesia and was in a state of higher stress / sympathetic tone, which also has an impact on heart rate and electrophysiological features such as QT duration and T-wave morphology. Breathing rate was determined by doing a fast Fourier transform and determining the dominant frequency of the signal of lead 32, *i.e*. the one located towards the left leg of the rabbit. The provided BSPM recordings themselves were done 3 days after the CT imaging, with the vest ensuring the same electrode locations.

To analyze the BSPM data, the recorded signals were filtered using a high-pass filter (type: Butterworth; order N = 4; cutoff = 1.2Hz) to remove breathing artifacts, and a band-stop filter (type: Butterworth; N = 4; cutoff = [49.9, 50.1]Hz) to remove the 50Hz hum originating from mains power lines. We then averaged 30 heartbeats recorded between 10s and 20s, as the heart rate of roughly 2.85Hz during this period showed lowest variance (mean RR interval of 0.349 ± 0.02ms). All signal processing was done in Matlab R2017a (The Mathworks Inc., Natick, MA), with the *filtfilt* function being used in the filtering part.

### Geometry generation

**Fig 2:**
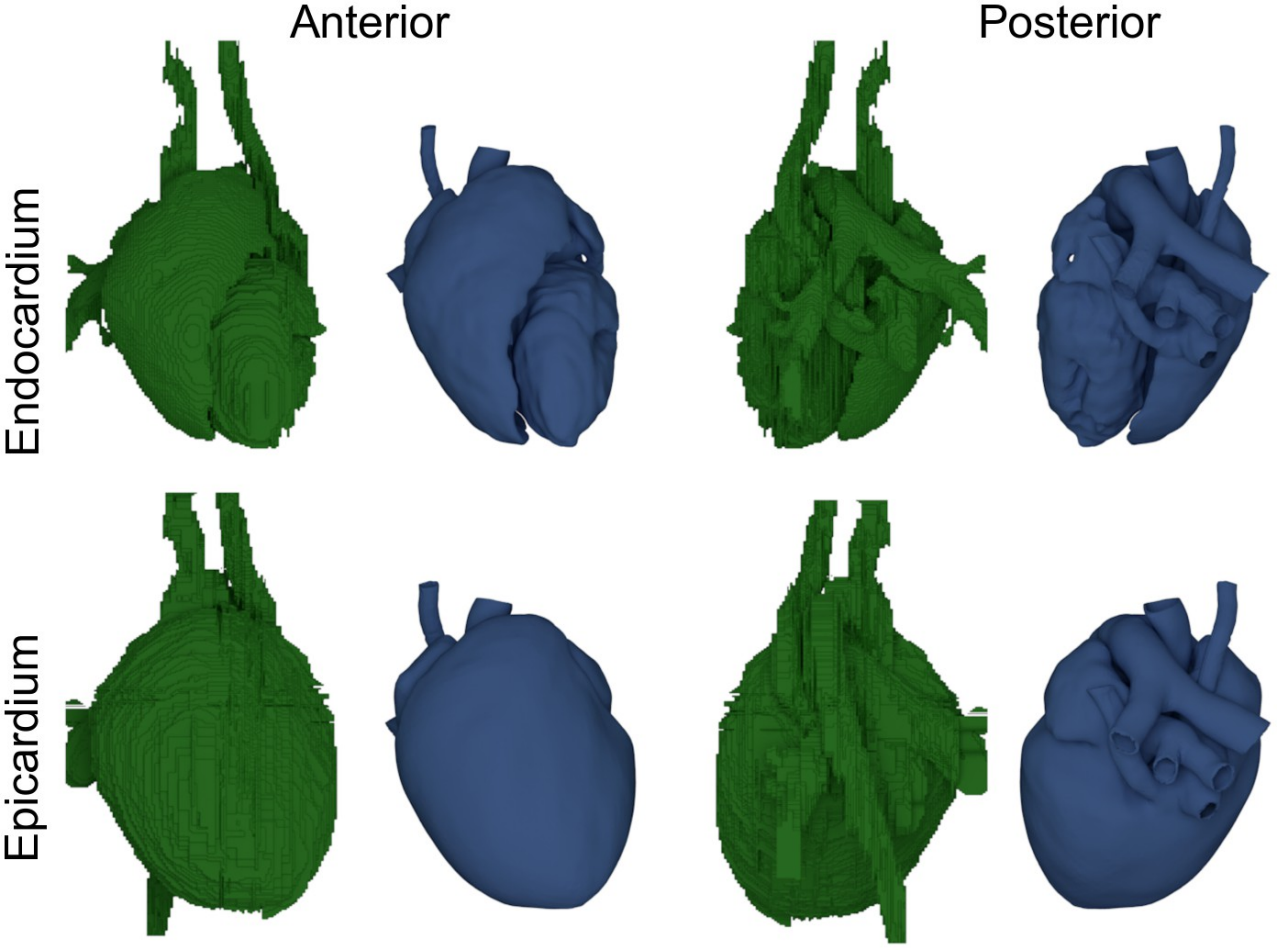
Surfaces of the initial segmented heart (green) and the resulting processed smoothed surfaces (blue). Anterior and posterior view of the initially segmented surfaces (using Seg3D2 [17]) of endo-, and epicardium and the resulting retopologized smooth surfaces (using Blender [18] and InstantMeshes [19]).

Based on the CT image data, tetrahedral meshes of heart, liver, lungs, bones, cartilage, and stomach were created. All organs were initially segmented using SEG3D2 [17], resulting in a voxel mesh each. The meshes were then imported into Blender (version 2.83 LTS) [18]. There, all meshes were retopologized and potential errors in the segmentation, such as holes and overlaps, were fixed manually. A comparison between the initially segmented and processed surfaces of the heart can be seen in Fig 2, or in more detail in S1 Video. This procedure of retopologizing was especially difficult for the atria. Here, due to the thinness of the atrial wall, which was occasionally below the image acquisition resolution, the geometry was modeled based on adjacent features to the best of our understanding. As papillary muscles could not be processed by the later described algorithm to determine the Purkinje network, they were removed. To ensure good tetrahedral mesh quality later on, the resulting fixed triangle surface meshes were afterwards once more retopologized using InstantMeshes [19]. The surface meshes were then used to create tetrahedral meshes using GMSH (version 4.8.4) [20]. The material classes of the published mesh are given in Table 1.

**Table 1:** Material classes and conductivities used to calculate the lead field matrix.

	Material class	*σ*_*it*_	*σ*_*il*_	*σ*_*et*_	*σ*_*el*_
Aorta	60			0.25	
Pulmonary artery	61			0.25	
Left vena jugularis	62			0.25	
Right vena jugularis	62			0.25	
Post vena cava	62			0.25	
Left atrium	33	0.0193	0.174	0.236	0.625
Right atrium	32	0.0193	0.174	0.236	0.625
Left ventricle	31	0.0193	0.174	0.236	0.625
Right ventricle	30	0.0193	0.174	0.236	0.625
Fat	2			0.035	
Bones	3			0.02	
Blood	9			0.7	
Cartilage	14			0.15	
Liver	20			0.02	
Lungs	17			0.03	

All isotropic conductivities of the organs were taken from Keller *et al*. [21], which in turn used the values measured by Gabriel *et al*. [22]. The respective intra- (*σ*_*i*_) and extracellular (*σ*_*e*_) conductivities of the heart were taken from Clerc *et al*. [23], with subscript ‘t’ depicting the conductivity in transverse and ‘l’ in longitudinal direction. All conductivities given in S/m.

**Fig 3:**
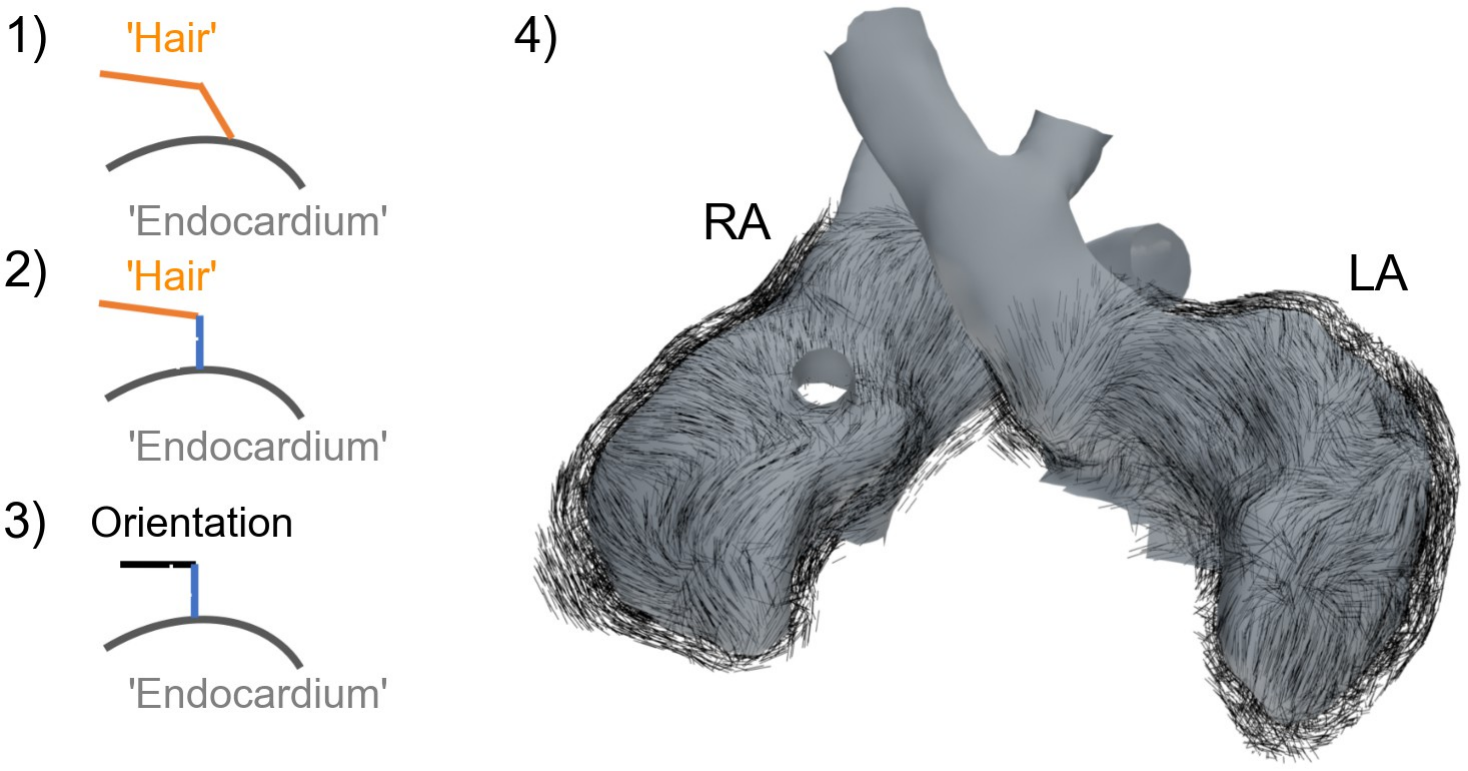
Workflow to generate atrial myocyte orientation. Using a hair particle system with two segments (2 line elements) in Blender [18], the hair was groomed to visually recreate the myocyte orientation published by Kharche *et al*. [25], step 1. Then the segment connected to the endocardial surface is corrected to be in normal direction, step 2. By assuming that the myocyte sheet direction is in parallel and the sheet-normal direction is in normal direction in respect to the endocardial surface, we can correct the second segment such that it is orthogonal to the other two directions, step 3. Finally, the segment connected to the endocardial surface is removed, leaving the second segment depicting the myocyte orientation (not defined for vessels), step 4.

Myocyte orientation for the ventricles was set based on the rule-based approach presented by Bayer *et al*. [24], using the published values of *α*_*Endo*_ = 40°, *α*_*Epi*_ = −50°, *β*_*Endo*_ = −65° and *β*_*Endo*_ = 25°. Regarding the atria, the data published by Kharche *et al*. [25] was used as a reference. Therefore, growing from the endocardial surface, a hair particle system with each strand comprising two segments (i.e 2 line elements) was created in Blender, which was then ‘combed’ (a functionality within Blender) as to visually recreate the aforementioned orientation. The segment connected to the surface was then removed and the remaining segment was corrected to be parallel to the nearest face of the endocardial surface (under the *a priori* assumption that the myocyte sheet-normal direction is orthogonal to endocardium) and then normalized to be used as myocyte orientation, see Fig 3 for an illustration of the algorithm. For all other organs, no preferential cell orientation was specified.

### Electrophyisological modeling

#### Excitation propagation

To simulate electrical excitation and wave propagation across the tissue, we used the framework *acCELLerate* [26] with custom extensions allowing us to use tetrahedral mesh-based geometries. Transmembrane voltage (*V*_*m*_) across the tissue was governed by the monodomain model, a reaction-diffusion equation:

$$\begin{array}{c} \nabla \cdot \left(\sigma \nabla V_{m}\right)=\beta (C_{m}\frac{dV_{m}}{dt}+I_{ion}) \end{array}$$ (1)

where ***σ*** is the conductivity tensor, *β* = 140, 000 m^−1^ the myocyte surface to volume ratio, *C*_m_ = 3.1 × 10^−4^ *μ*F the cell membrane capacitance, and *I*_*ion*_ is the sum of ionic currents across the membrane (*i.e*. currents between intra- and extracellular space).

Based on Schalij *et al*. [27], to achieve a conduction velocity of 225 mm/s in transversal and a conduction velocity of 610 mm/s in longitudinal direction, the monodomain conductivities *σ*_*t*_ and *σ*_*l*_ were set to 0.016 S/m and 0.0392 S/m, respectively. *I*_*ion*_ was calculated using the myocyte electrophysiology model by Mahajan *et al*. [28], which was slightly modified as described later, comprising ordinary differential equations. The finite element method was used for spatial discretization of the monodomain equation. Operator splitting was employed to separately integrate the myocyte models and monodomain equation in time. The second order Crank-Nicolson scheme was used for temporal discretization of the monodomain equation [29]. For all gating variables in the myocyte model, the Rush-Larsen method was implemented and for all other variables the explicit Euler method was used. The simulations were discretized with 2 × 10^−5^ s in time.

#### Calculation of body surface ECG

The forward problem of the ECG, *i.e*. the calculation of body surface potentials based on *V*_*m*_ gradients computed using the bidomain theory, was solved considering a lead-field (LF) matrix approach. The LF matrix was calculated as published by Potse *et al*. [30], with the extracellular conductivities given in Table 1, based on the work by Keller *et al*. [21] and Clerc *et al*. [23]. As we were only interested in the potential at specific points on the torso surface (the 32 locations of the electrodes), using a LF matrix instead of solving a full bidomain simulation for the complete torso offered significant advantages in terms of computational cost, especially because we used an iterative optimization procedure necessitating many ECG calculations (see later).

#### Ventricular activation sequence

The ventricular activation sequence was calculated using the algorithm presented by Kahlmann *et al*. [31]. The respective algorithm optimizes the node density, maximal tree height, conduction speed, and location as well as temporal activation offset of Purkinje-muscle junctions of a Purkinje network. For each iteration of the optimization, based on the resulting stimulation locations and temporal offsets, a fast marching algorithm was used to determine activation times throughout the ventricles. Then, using a predefined action potential snippet translating activation time into *V*_*m*_, a forward calculation was performed using the aforementioned LF matrix. The resulting QRS-Complex of the lead signals was then quantitatively compared to measured ones using a root mean square error (RMSE) function. As this method was originally designed to optimize the activation sequence of a human ventricle based on a 9-lead ECG, the algorithm was modified in the following parts. The underlying human AP snippet was replaced by one extracted from a monodomain simulation using the Mahajan *et al*. rabbit ventricular myocyte model [28]. Additionally, instead of assuming a uniform conduction velocity, the conduction velocity within the fast marching algorithm was set to be 225 *mm*/*s* in transversal and 610 *mm*/*s* in longitudinal direction, *i.e*. the same as in the monodomain simulation (based on [27]). Further, the RMSE function was extended to include all recorded leads:

$$\begin{array}{cc} & Lead_{i}^{sim}=Signal_{i}^{sim}-wct^{sim}\\ & Lead_{i}^{mes}=Signal_{i}^{mes}-wct^{mes}\\ & wct=(Signal_{5}+Signal_{7}+Signal_{31})/3\\ & error=\sum_{i}^{31}\sqrt{(Lead_{i}^{sim}\cdot f-Lead_{i}^{mes}{)}^{2}} \end{array}$$

with:

$$\begin{array}{cc} & f=max\left(Lead_{13}^{mes}\right)/max\left(Lead_{13}^{sim}\right), \end{array}$$

where *Signal*_*i*_ is the simulated (*sim*) or measured (*mes*) signal at electrode *i*, *Lead*_*i*_ the signal in respect to the Wilson central terminal (*wct*), which is calculated based on the signals on the right arm (*Signal*_5_), left arm (*Signal*_7_), and left leg (*Signal*_31_), and *f* a scaling factor calculated based on the occurring maxima in simulated and measured *Lead*_13_. The scaling factor was needed as simulated and measured signals showed a difference in amplitude.

#### Ventricular electrophysiological heterogeneity

**Fig 4:**
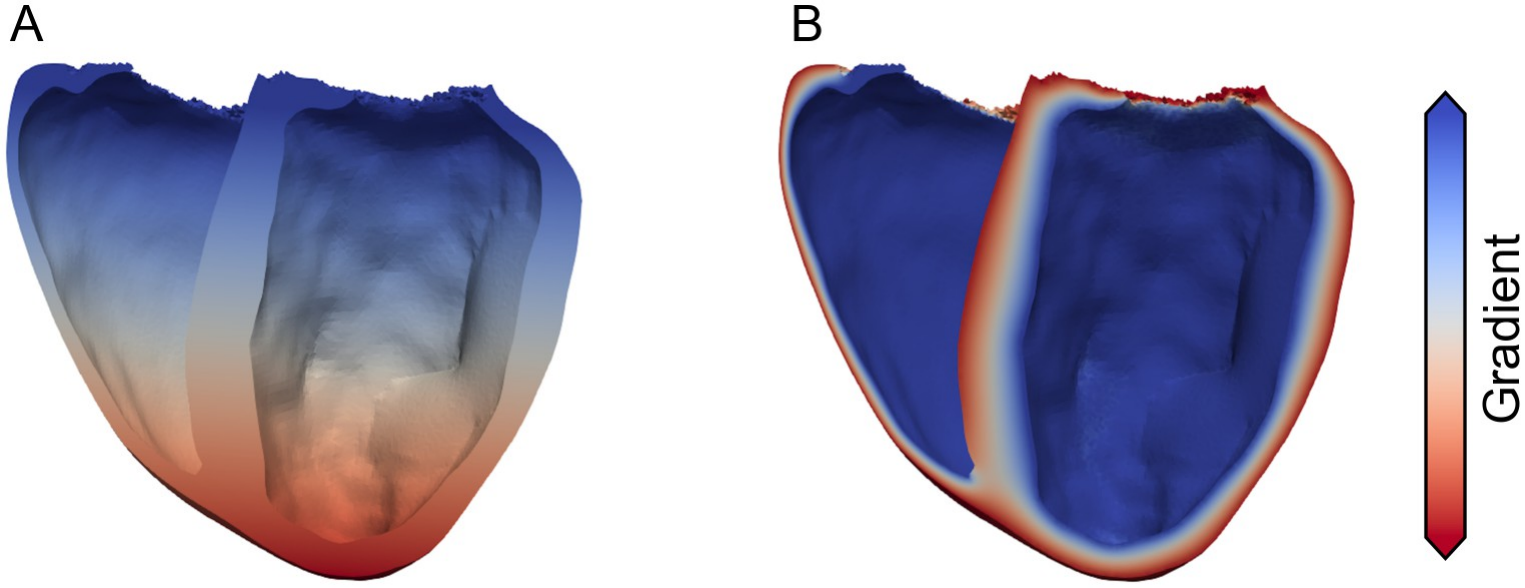
Transmural and apico-basal gradients. Transmural (A) and apico-basal (B) gradient used to define different gradients in APD. The gradients were flipped for respective APD lengthening; meaning that for example for a shortening in APD from base towards apex, blue represents 0 ms and red the APD shortening; for a shortening towards the base (*i.e*. a lengthening towards apex), blue would represent the APD shortening and red 0 ms.

**Fig 5:**
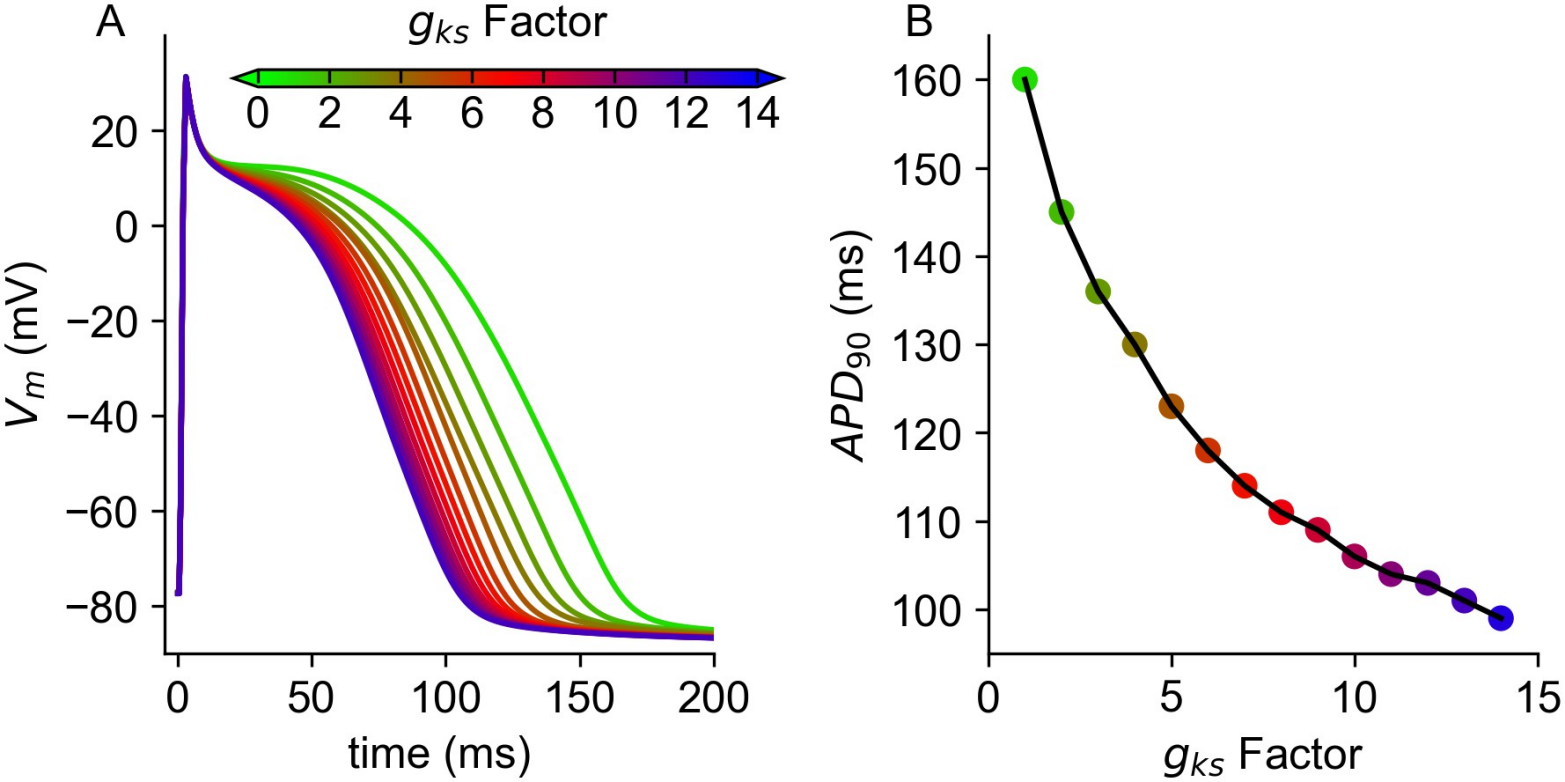
Action potential and APD_90_ in relation to *g*_*ks*_ conductivity. Resulting action potentials (A) in relation to different factors of *g*_*ks*_ (gksFac) and resulting APD at 90% repolarization.

Measured data as well as computational studies have shown that in the healthy heart gradients in APD exist from apex to base and from endocardium to epicardium [2, 12, 32, 33]. In order to be able to compare the resulting simulated ECG to the measured one, the used ventricular rabbit single cell model by Mahajan *et al*. [28] had to be modified. Specifically, *g*_*kr*_ was increased by 100%, such that the resulting APD at 90% repolarization at 2Hz was as published by Idriss *et al*. [32], i.e. 177ms. Subsequently, the model was stimulated at the same frequency as the one extracted from the measured data (2.85Hz) until it reached limit cycle. Additionally, in the whole ventricle setup, a transmural heterogeneity in *g*_*to*_ (based on Fedida *et al*. [33]) was introduced, such that the conductance in the channel was 27% higher in the epicardial region than in the endocardium. This configuration was then used as the starting point to examine the feasibility of different combinations of apico-basal and transmural heterogeneities in the APD distribution. As a first step, two respective gradients were calculated, with one going from base to apex and the other one from endocardium to epicardium, see Fig 4. The investigated differences in APD ranged between −60ms and + 60ms. It should be noted that for heterogeneities with a lengthening in APD, the gradient was flipped to obtain ECGs with a comparable QT-Time, i.e the longest APD occurring throughout the ventricles was always 177ms. The resulting distribution of APD was then realised by adjusting *g*_*ks*_ accordingly. The correlation between APD and *g*_*ks*_ was calculated beforehand and can be seen in Fig 5. The models were then grouped with a variance of 5% (regarding the *g*_*ks*_ conductance), and paced into limit cycle once more prior to the whole heart simulations. As the model showed characteristics of alternans for APD shortening of more than 65ms, heterogeneities where the maximal change in APD surpassed 60ms were not examined. Using a temporal discretization of APD differences of 5ms, this resulted in a total of 364 variations/simulations.

## Results

### ECG recordings

The overall procedure of shaving the anesthetized rabbit, putting on the vest, and connecting the individual electrodes took less than 15 minutes. The signal-to-noise ratio (SNR) of the recorded signal averaged across all leads was 5.01±1.16 (mean±std), with the noise being estimated as the difference between the signals before and after filtering. A comparison between the respective 30 measured heart beats and the resulting averaged beat for each ECG-Lead is depicted in S1 Fig. The resulting signal amplitude is comparable to values found in literature [34, 35].

### Geometry

**Fig 6:**
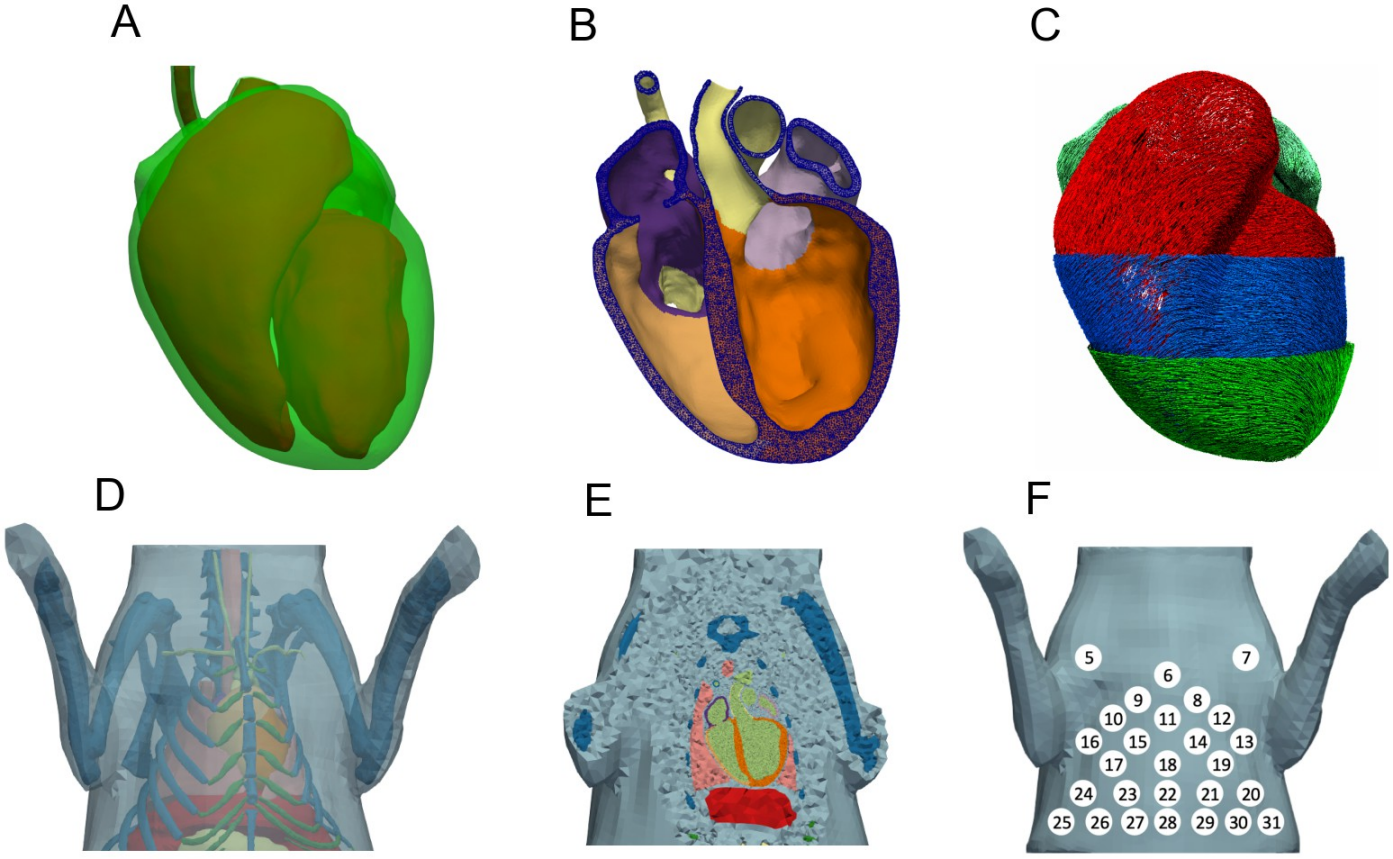
Heart and Torso surfaces and geometry. A: Surfaces of the endocardium (red, solid) and epicardium (green, transparent) of the heart. B: Open cut through meshed (tetrahedral) geometry of the heart. C: Representation of cardiomyocyte orientation of the heart, with the ventricles being visualized on endo-, mid-, and epicardial areas. D: Surface of the rabbit torso and all its organs (Light-blue: fat, Dark-blue: bones, Dark-green: cartilage; Salmon: lungs, Red: liver; Light-green: blood, Light-yellow: stomach). E: Open cut through the meshed geometry of the torso. F: Location of the ECG-Leads on the ventral side of the rabbit vest (5–31).

Fig 6A shows the resulting surface of the heart and Fig 6D the surfaces of the torso itself and all organs. The resulting meshed geometry of the heart (Fig 6B) comprises 3.3 × 10^6^ tetrahedral elements with an average edge length of 0.267 ± 0.05mm. Out of these, 2.5 × 10^6^ are part of the ventricles, 0.5 × 10^6^ part of the atria, and the remaining 0.3 × 10^6^ part of blood vessels. But, the surfaces of the heart could easily be retopologized once more to achieve a different desired resolution. The generated myocyte orientation of the ventricles and atria are shown in Fig 6C, with the former being created using a published and validated algorithm [24] and the latter being manually ‘groomed’ based on measured data [25] as previously described. Fig 6D depicts the surface of the rabbit torso itself and all its organs. The resulting meshed geometry, comprising 10.5 × 10^6^ tetrahedral elements (out of which 3.3 × 10^6^ are part of the heart) is shown in Fig 6E. It should be noted that during the generation of the surfaces, a small layer of fat surrounding the heart was included. This was primarily done to make it easier to substitute the heart with one having a different surface mesh resolution, without needing to adapt other surfaces as well. Further, it should be made clear, that everything labeled as fat in the torso model is a mixture of fat and muscle since the differentiation between the two was not clear in the CT image data and thus they were treated as one tissue class. The position of the electrodes, on the surface of the torso geometry, (extracted from CT image data) can be seen in Fig 6F. Not shown are electrodes 1–4, which are located on the back of the rabbit, and electrode 32, which was placed further down towards the left leg to be used as a reference when looking at individual lead traces.

### Electrophysiology

#### General

As previously described in the method section, to compare measured and simulated signals, the Wilson central terminal was used as reference potential for both, and the latter was scaled by a factor *f* of 0.44 ($f=max\left(Lead_{13}^{mes}\right)/max\left(Lead_{13}^{sim}\right)$).

#### Depolarization

**Fig 7:**
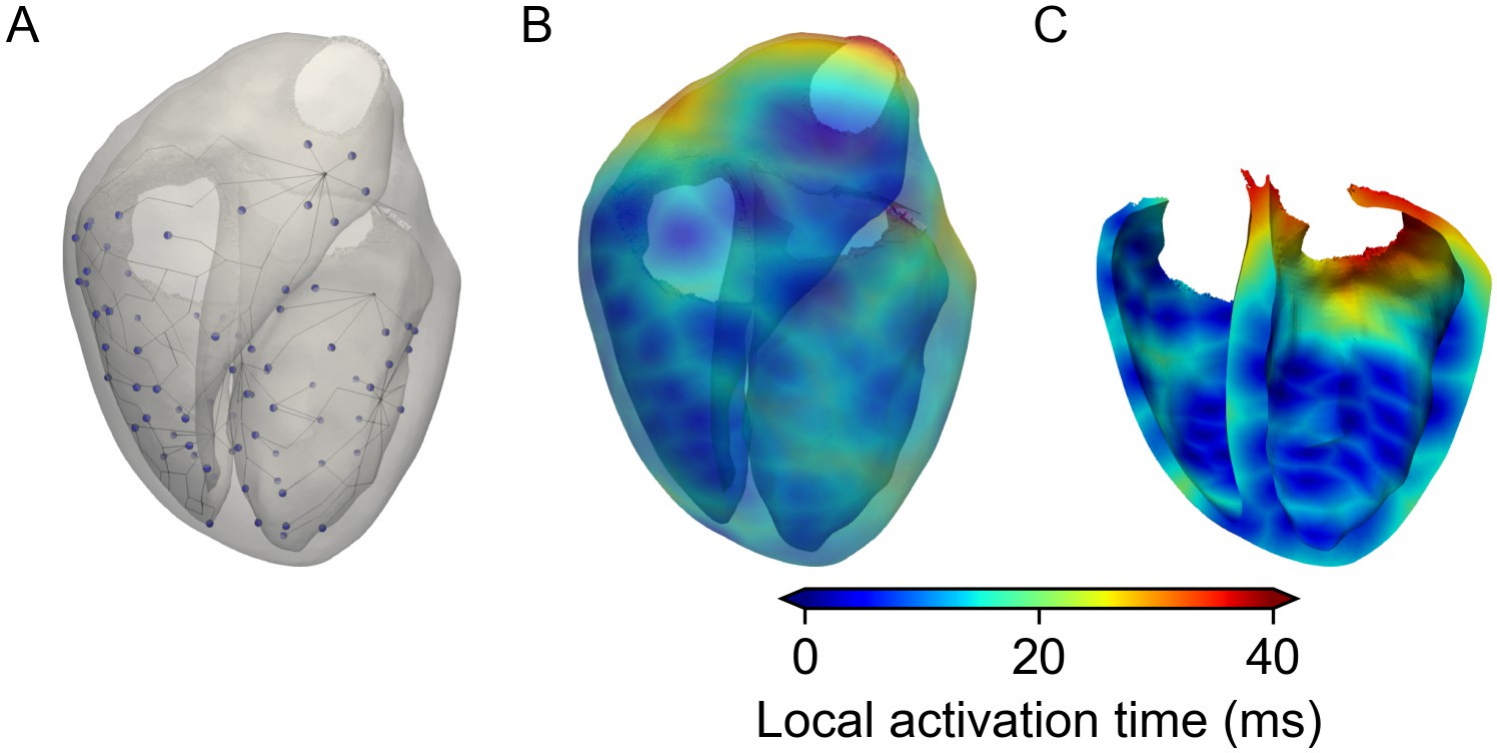
Optimized activation sequence and local activation times. A: Resulting optimized Purkinje tree used to generate activation times within the ventricles. B: Local activation times throughout the ventricles. C: Cut view of the ventricles showing the initial activation at the septum. Due to the small size of the ventricles and the high velocity within the Purkinje network of 3655mm/s, all stimulation points activate roughly synchronously.

The depolarization time was defined as the time between first stimulation (0 *ms*) and the time where all nodes in the ventricle were excited (50 *ms*), with the right ventricular outflow tract being activated last, see Fig 7B. Optimization of the activation sequence resulted in an average RMSE between measured and simulated signal of 0.074 ± 0.04 mV/ms across all leads. The smallest RMSE of 0.0267 mV/ms can be seen for *Lead*_5_ and the largest (0.16 mV/ms) for *Lead*_10_. In general, leads closer to the heart showed a more similar QRS-Complex and consequentially smaller error than those further away from the heart. In terms of parameters, the optimization yielded three root points (from which the Purkinje tree branched out of) at the left ventricular side of the septum and two roots in the right ventricle, located opposite to each other on the posterior and anterior side, see line representation in Fig 7A. The conduction speed of the network itself was 3655 mm/s with a rather low spacing of approximately 2.5 *mm* between excitation points (Purkinje-muscle junctions). Further, for all leads the simulated QRS-Complex seems to be a good fit in terms of duration. In terms of amplitude, there are some exceptions, *i.e. Leads*_9,19,15,17_, where the S-peak amplitude is visibly lower in the simulation compared to the measured one. In contrast, *Lead*_8_ is the only lead where the simulated amplitude of the S-peak clearly surpasses the measured one.

#### Repolarization

**Fig 8:**
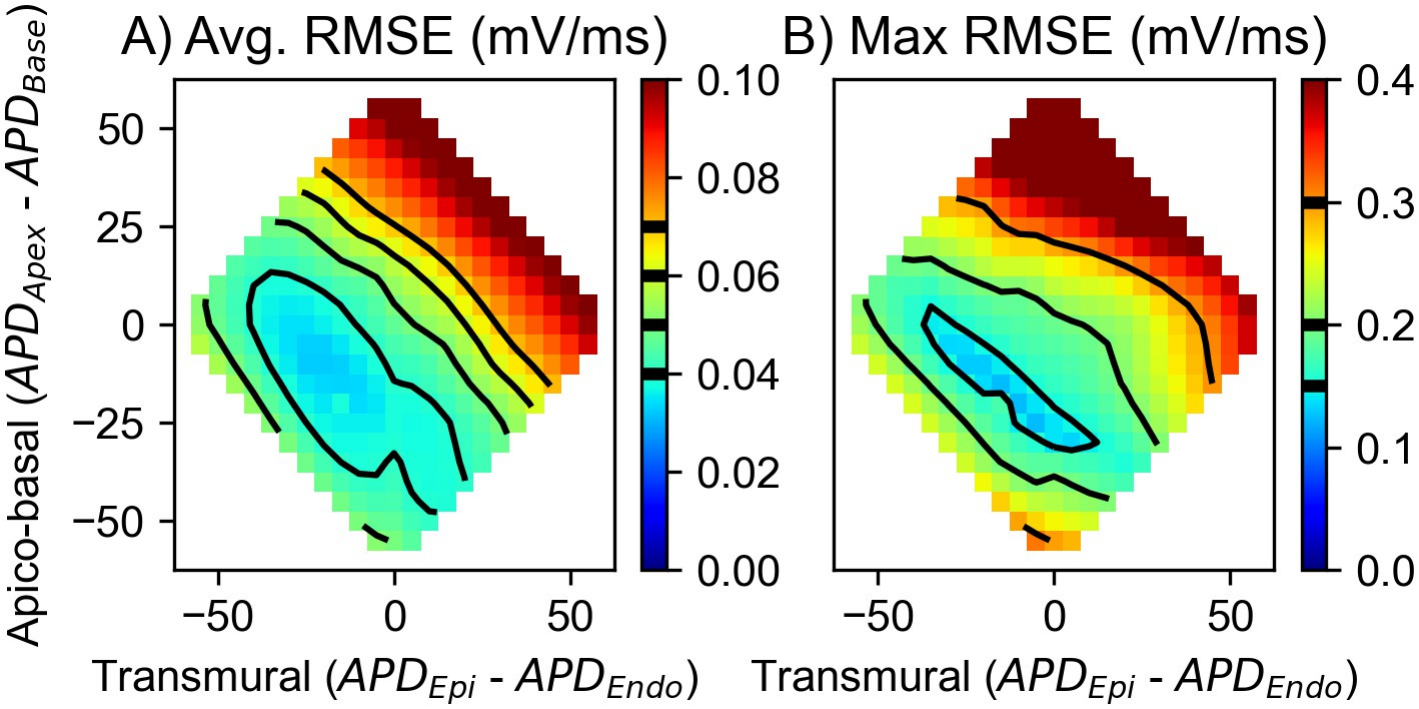
Resulting mean and maximum RMSE of all leads. Resulting mean error of all leads (A) and maximum occurring RMSE error across all leads (B). As the mean error in (A) on its own does not provide a clear global minimum across all combinations of APD gradients, we looked further into maximum occurring error in (B).

**Fig 9:**
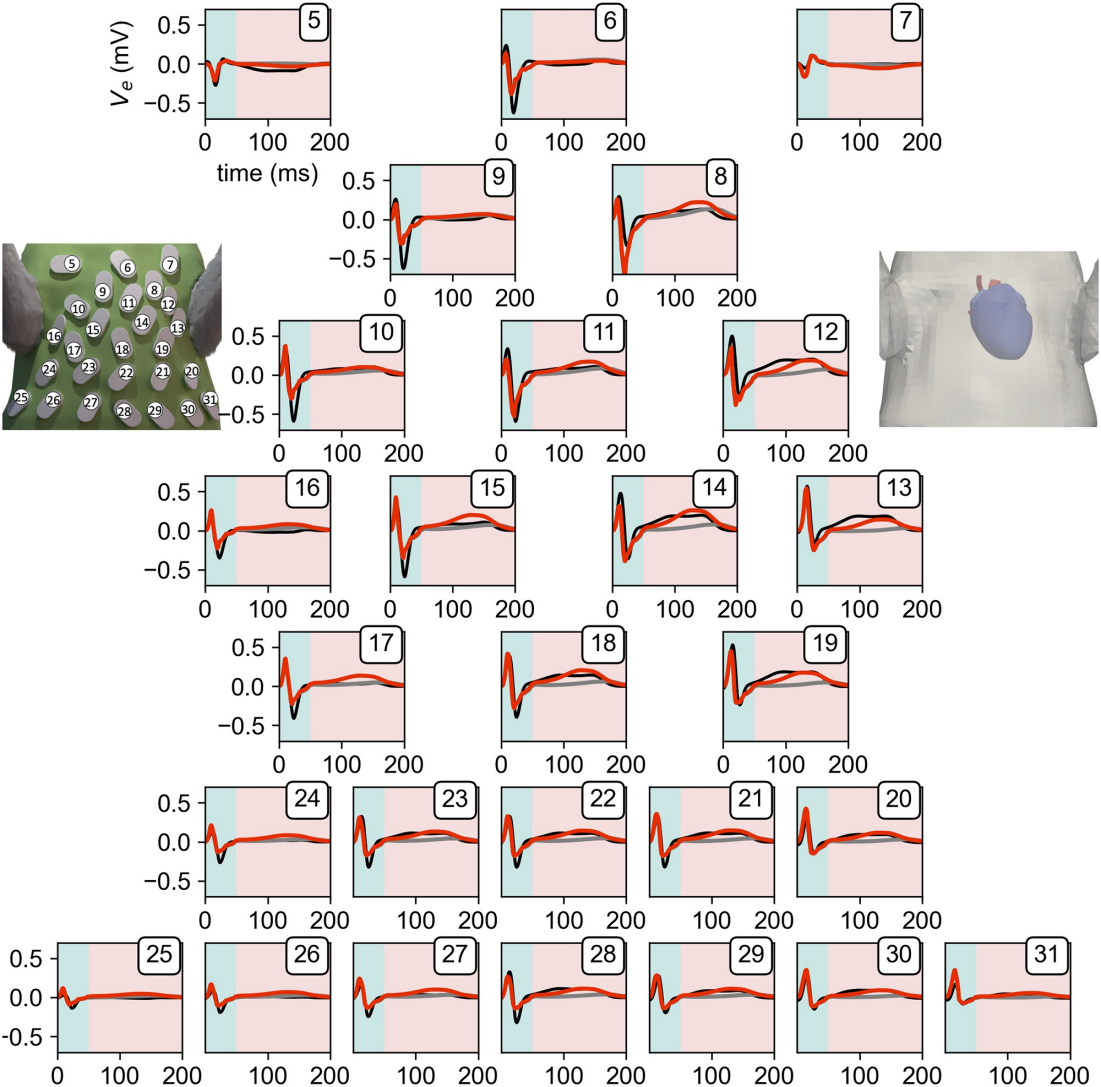
Resulting measured and simulated lead signals. Measured (black), simulated without any gradient (gray), and best simulated gradient (red) signal of body surface potential traces for *Lead*_5_–*Lead*_31_ (*Lead*_1_–*Lead*_4_, *Lead*_32_ not shown). The simulated traces where scaled as described in the methods section. Turquoise background indicates the QRS-Complex (1–50 ms) and salmon the T-Wave (50–200 ms). Each subplot is labeled by the respective lead number on the top right corresponding to its placement seen in the left image of the ECG-vest. The displayed simulated trace incorporates a gradient in APD of a shortening towards the apex of 20 ms and a shortening towards the epicardium of 15ms. This results in an overall average RMSE of the leads during the QRS-Complex of 0.074 mV/ms and 0.038 mV/ms during the T-Wave part.

The repolarization of the ventricles and the subsequent gradients in *V*_*m*_ are the electrical cause of the T-Wave (50–200 ms) in the ECG. We investigated various apico-basal and transmural APD gradients ranging from −60 ms to +60 ms in each direction and their respective combinations. Our findings show that, as expected, both play a role in the generation of a physiological T-Wave, with the apico-basal being the more impactful one. Fig 8A shows the resulting average RMSE across all leads for all combinations of APD gradients. One can see that combinations of APD shortenings from base towards apex and from endocardium towards epicardium produce the smallest average RMSE across all leads. But solely looking at the average RMSE yielded no global minimum as multiple combinations resulted in errors ranging between 0.035 and 0.04 mV/ms. Thus, we further looked at the maximum occurring RMSE across all leads, see Fig 8B. By doing so we, could narrow down that any combination of shortening gradients, which in combination adds up to 40ms seem to be valid and does not result in single leads worsening significantly with the average RMSE staying the same. Subsequently, the combination which showed the smallest average and smallest maximum RMSE was the one with a shortening towards the apex of 20ms and a shortening towards the epicardium of 15ms. Fig 9 shows these optimized best traces (red) of all leads in comparison to measured ones (black) and simulations without the inclusion of any gradient additional to the transmural one in *g*_*to*_ (gray). The optimized trace of the T-Wave had an average RMSE of 0.038 ± 0.024 mV/ms, which is half of the mean error measured during the QRS part of the ECG, and a maximum RMSE of 0.095mV/ms. Interestingly, there seem to be two kind of deviations between measured and simulated signals, one being a difference in the early and the other a difference in the later part of the T-Wave. The first one can be seen in *Lead*_12_,*Lead*_13_,*Lead*_14_, and *Lead*_19_ and the second in *Lead*_11_,*Lead*_15_, and *Lead*_17_. In general, all respective leads show a similar combined affinity towards changes in both APD gradients, see S2 Fig. Most notably, *Lead*_6_ seems to be predominantly influenced by the apico-basal heterogeneity gradient and *Lead*_7_ by the transmural one. In S3 Fig, we then looked at how the different heterogeneities affect the maximal amplitude and subsequent direction of deflection of the T-Wave. The amplitude in the lower row (*Lead*_20_–*Lead*_31_), shows a very similar behavior, being mainly dependent on the transmural gradient. The next two rows (*Lead*_13_–*Lead*_19_) depict a similar pattern, as the leads on the left side of the body are mainly influenced by the transmural gradient with the influence of the apico-basal gradient gradually increasing towards the right side of the body.

## Discussion

### Summary

Within this manuscript, we have presented a self-fabricated 32-lead ECG-vest used to record the BSPM of a rabbit. Then, using measured CT image data of the same rabbit, we created a geometrical model of the *in-vivo* rabbit heart and torso. The combination of measured BSPM and generated geometrical model enabled us to parameterize the ventricular activation sequence needed to recreate the QRS part of the ECG. Subsequently, we investigated the feasibility of different physiological apico-basal as well as transmural APD gradients within the ventricles based on the measured T-Wave part of the ECG.

### Vest and ECG recordings

Certainly, increasing the number of electrodes could aid capturing the field distribution on the torso surface more accurately. But we were limited, due to the size of the electrodes and the corresponding electrode holders, as seen in Fig 1. Looking at our measured traces we can see that the variance between neighbouring electrode signals is already quite low, especially for the electrodes located towards the lower part of the torso. A different approach would be to use electrodes designed to be used for infants, as published by Zhang *et al*. [10]. Here, they were able to place forty to sixty electrodes uniformly across the torso surface. Yet, this method relies on manually placing the electrodes, and has potentially less consistency across multiple measurements and/or different rabbits. Ideally, one would combine the best of the two and redesign our vest to be used with a different measurement system which electrodes need less space.

### Geometry

As previously stated, due to the thinness of the atrial wall, which was occasionally below the image acquisition resolution, the atria were the most difficult part to segment. Especially, as the iodine solution to enhance contrast in the CT was administered *via* one auricular vein, the right *V. jugularis* and some upper parts of the right atrium showed lower contrast than those in proximity of the left *V. jugularis*. The most obvious way to improve this would be to use a CT with a higher resolution, and/or trying to administer the contrast agent *via* both auricular vein (*i.e*. both ears) simultaneously to ensure even distribution in left and right *V. jugularis* and thus eliminating the contrast gradient within the right atrium. Nonetheless, our geometrical model of the heart itself already shows distinct advantages over other available models. For one, it is the only *in-vivo* model to include the atria themselves. Further, it is an accurate representation of the heart in its *in vivo* state. Compared to the ventricular models by Vetter *et al*. [5] and Bishop *et al*. [6] our model depicts much thinner ventricular walls. This is due to the fact that these models, as they were created based on extracted fixed hearts, lack diastatic pressure. That being said, it should also be pointed out that our final geometrical model does not include any information about the papillary muscles. We have added a segmented endocardial surface to the online repository, which includes the papillary muscles, but not to an extent and detail as shown in the Bishop *et al*. [6] and Stephenson *et al*. [8] models. Regarding atrial myocyte orientation, algorithms to generate myocyte orientation do exist, however they require the definition of specific landmarks in the atria, which were originally defined according to the human atria, see Rooney *et al*. [36] or Wachter *et al*. [37]. As the orifices of human and rabbit atria differ, they could not be applied here but potentially could be adapted. Yet, one would need to validate such algorithm against measured data, ideally from the same rabbit. This could be done by sacrificing the rabbit, fixing the heart and then either extract the information from slices [5, 6], micro-CT [8], or from diffusion tensor MRI [7, 25]. But one would potentially need to develop an algorithm which takes into account deformation as well as volume changes of the tissue itself, as for example done in Wülfers *et al*. [38]. This would also potentially solve the lack of information currently included in terms of endo- and epicardial separation and criss-cross pattern which one would expect in the atria. About the torso geometry, here our geometry assumes everything that was not respectively segmented to be one tissue type (fat). This is of course not accurate, as it should be more of a mixture of fat and muscle. A better differentiation between the two could be achieved by basing the geometry itself on MRI imaging. But—speaking from our experience—within such a data set, the segmentation of the heart itself would prove difficult.

### Electrophysiology

#### General

The fact that a scaling factor (0.44) smaller than 1 was needed to adjust the simulated ECGs to the measured ones indicates that a potential dampening factor was not sufficiently incorporated in the simulations. The scaling factor itself could also be incorporated by scaling of the extracellular conductivities, as the bidomain model is a linear mathematical problem. As such, the conductivity which scaling almost translates one to one on the resulting signal amplitude is the intracellular and extracellular conductivity of the heart used in the forward model, *i.e*. lead field matrix calculation. The main reason for this is most like the overall size of the rabbit torso and the close location of the heart to the body surface itself. Unfortunately literature values for this specific conductivity differ greatly and have been the focus of multiple publications. The one used within this work by Clerc *et al*. [23], was chosen as it resulted in the best scaling factor, even though it was originally measured in calf hearts.

#### Depolarization

The optimization of the Purkinje tree resulted in an activation sequence which is largely able to reproduce its physiological counterpart. Especially, the Q and R-Peak of the QRS-Complex show a high similarity between measured and simulated traces and it can be deducted that the initial stimulation points and activation times are a good fit. Some room for improvement can be seen regarding the S-Peak in some leads. As can be seen in *Lead*_9_, *Lead*_10_ and *Lead*_15_, where the resulting simulated amplitude is considerably lower than the measured one. Due to the location of said electrodes, *i.e*. atop the right ventricle, in combination with the local activation times (see Fig 7B.) one can assume this is either due to the lack of regional differences or misalignment of the myocyte orientation at the right ventricular outflow tract. Here a similar approach to as proposed by Zhang *et al*. [10], to validate the activation times could further help with illuminating the underlying reason.

#### Repolarization

Compared to literature, our results clearly support the hypothesis of a coexisting apico-basal gradient of APD shortening towards the apex and a transmural gradient of APD shortening from endocardium to epicardium. The resulting best gradients in heterogeneity are in general in concordance with experimental values obtained by Mantravadi *et al*. [12], who reported a 17ms (at 2.38Hz) shortening in APD from base towards apex, and by Idriss *et al*. [32], who reported a shortening of 19ms (at 2Hz) from sub-endocardium to sub-epicardium. However, it should be noted that the latter observed an even larger gradient when looking at the endocardial and epicardial layer. The resulting gradient is also in agreement with our previous work by Keller *et al*. [2], where a similar modelling study was done regarding the human heart. However, in that study, the optimal transmural gradient also included a middle layer and thus resulted in a non-linear APD gradient from endocardium to epicardium. The work by Krishnamoorthi *et al*. [7] follows a similar approach of including a middle layer in their ventricular rabbit model, in addition to the same transmural gradient used within our work in *g*_*to*_, based on the measured data by Fedida *et al*. [33]. A comparison between our work and theirs is difficult though, as their shortest included AP is as long as the longest one within our model, *i.e*. 177 ms. Consequently, a simulated ECG including their conductivities would not fit well with our measured ECG data. In future, one could potentially investigate whether their gradient could be shifted such that the depolarization would align better with our T-Wave, similar to what we have done within this work. Yet, the impact of such a gradient is potentially amplified within their ventricular geometry, due to the previously mentioned inaccurate representation of the ventricular wall thickness. Additionally, when looking at the shortcomings of our simulated data, i.e a discrepancy in either the early or late part of the T-Wave, it is unlikely that they originate from a missing middle layer. The respective leads where there is a discrepancy in the early part of T-Wave morphology are located towards the left side of the torso and the ones where there is a discrepancy in the late part of T-Wave morphology are located towards the right side of the torso. As such, the underlying origin of the former, is most likely a lack of early regional differences, *i.e*. during phase 1 and 2 of the AP in the left ventricle and the latter could be explained by the fact that we assumed the gradient in the right ventricle to be same as in the left, which needs not be the case.

## Outlook and conclusion

In general, our geometrical model offers a vast amount of potential of follow-up projects. Based on the results of our presented study into the feasibility of ventricular heterogeneity gradients, one could potentially further investigate non-linear gradients (including ones that incorporate a mid layer) and potentially splitting the gradients for left and right ventricle. Additionally, although part of the measured data, a closer look at the P-Wave would most likely prove interesting and potentially also offer the possibility to validate the presented myocyte orientation. Though, one would need to implement the conductive pathways between left and right atrium such as the Bachman bundle and or other fast conductive tracks. In a more broad view, our openly available geometrical model gives other researchers the opportunity to incorporate it in their own research. Thus, it not only can be used to develop and verify algorithms with respect to the forward and or inverse calculation of the ECG but also presents a great opportunity to potentially translate insights gained from the rabbit to the human *via* the ECG.

As a conclusion, we were able to create a first openly available computational *in-vivo* whole heart as well as torso model of the rabbit. Further, we were able to design and build an ECG-vest, allowing us to record body surface potentials of said rabbit. Using those recordings, we were then able to optimize an activation sequence, recreating the Purkinje network, as well as investigate the viability of different apico-basal and transmural gradients in APD to reconstruct T-Wave morphology. Our investigation of different gradients and resulting ECG, shows that there is a coexisting apico-basal gradient with an APD shortening towards the apex and a transmural gradient with a shortening in APD from endocardium to epicardium. As such, our work supports the findings by Mantravadi *et al*. [12] and Idriss *et al*. [32], and consequently contradicts with findings by Cheng et al. [13] and our own previous work [14]. We hope that by making our underlying geometrical model and measured data publicly available, we can aid other researches in their computational research as well as bridge the gap of wet lab findings to their respective potential impact on the ECG.

## Supporting information

S1 Video Video comparing segmented and retopologized surfaces. Video of the segmented surface (green) using SEG3D [17] and the retopologized, smoothed surface (blue) using Blender [18].
(MP4)

S1 Fig Comparison of the underlying recorded beats and the subsequent averaged traces. Comparison between the 30 underlying recorded and filtered ECG beats and the resulting average signal trace for each lead. Temporal axis same as shown in Fig 9.
(TIF)

S2 Fig Individual average RMSE of *Lead*_5_–*Lead*_31_. Individual average RMSE of *Lead*_5_–*Lead*_31_ during the T-Wave window. The resulting average of all RMSE of the leads can be seen in Fig 8. The majority of the leads show a similar pattern in RMSE, with the exception of *Lead*_6_ and *Lead*_9_.
(TIF)

S3 Fig Maximum amplitude of the T-Wave for *Lead*_5_–*Lead*_31_. Maximum occurring amplitude during the T-Wave window, thus also showing the deflection direction of the T-Wave for the respective combinations of APD heterogeneity. The majority of leads show a dependency on both the transmural as well as the apico-basal gradient, with a few exceptions. Leads toward the lower middle of the torso are solely influence by the transmural gradient and *Lead*_9_,*Lead*_12_,*Lead*_13_, *Lead*_14_ solely by the apico-basal gradient.
(TIF)
