# Supplementary figures and images for: A computational model of rabbit geometry and ECG: Optimizing ventricular activation sequence and APD distribution
Source: PLoS One. 2022 Jun 30;17(6):e0270559. doi: 10.1371/journal.pone.0270559 (PMC9246225; doi:10.1371/journal.pone.0270559)

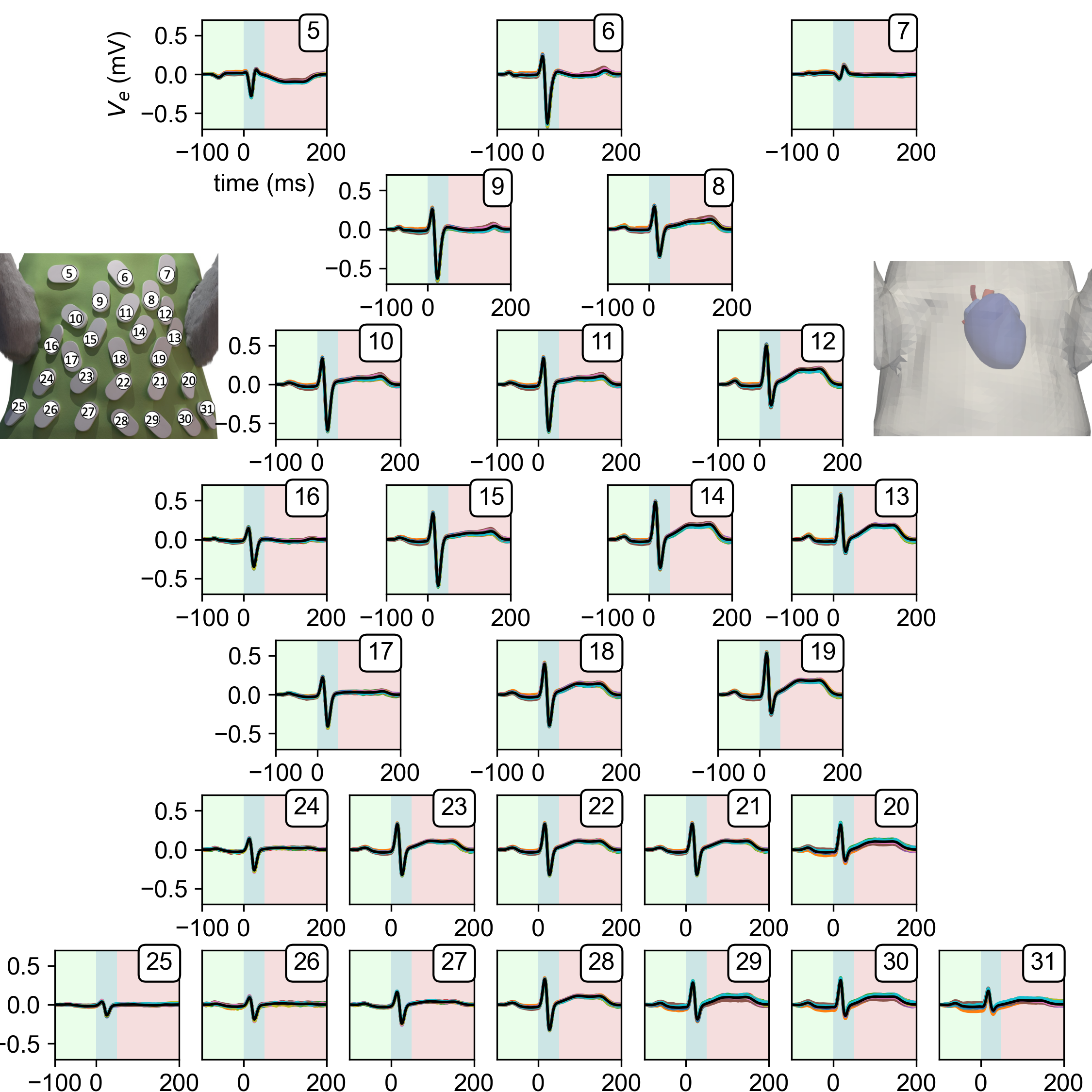

Supplement: S1 Fig — Comparison between the 30 underlying recorded and filtered ECG beats and the resulting average signal trace for each lead. Temporal axis same as shown in Fig 9. (TIF) [file pone.0270559.s002.tif]

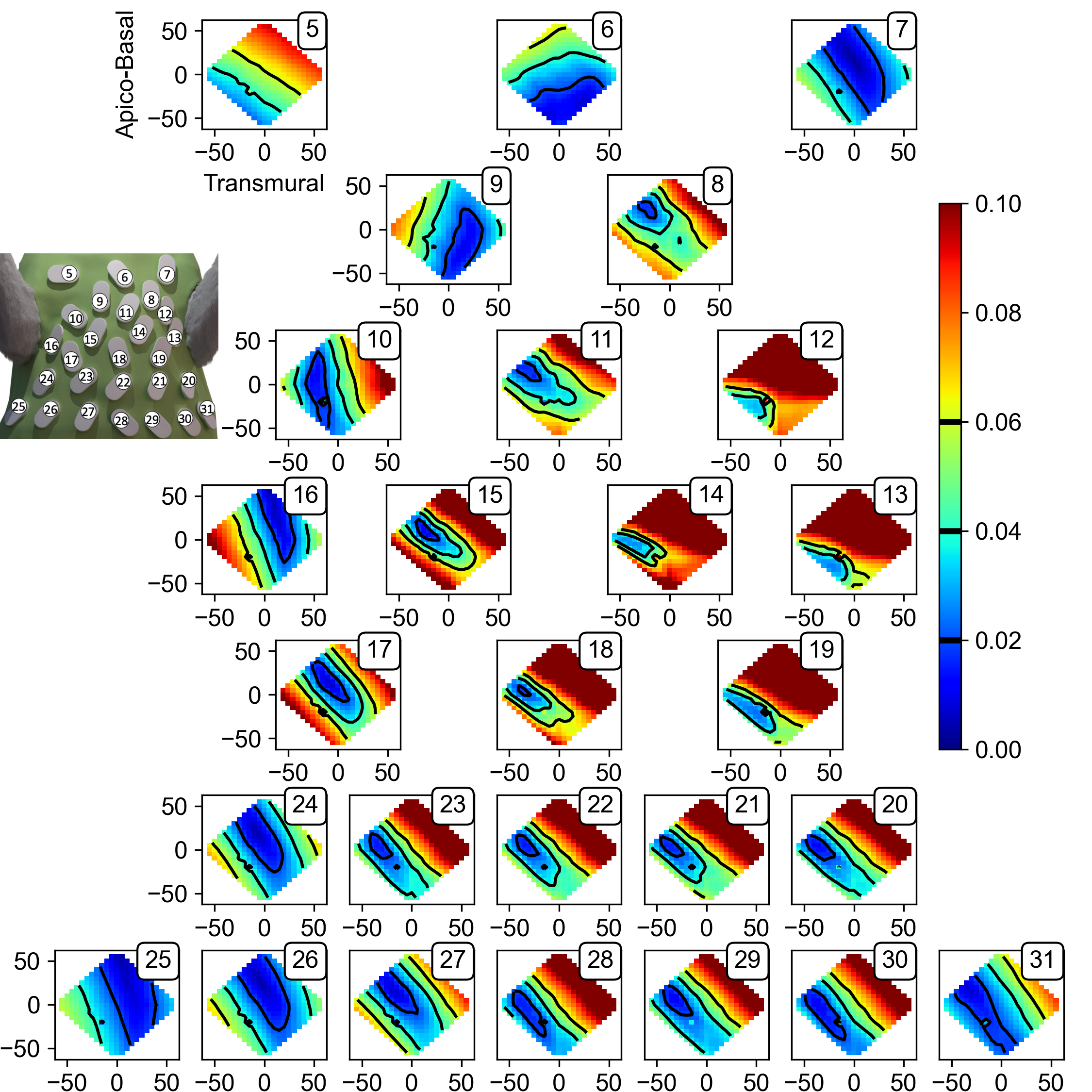

Supplement: S2 Fig — Individual average RMSE of Lead5–Lead31 during the T-Wave window. The resulting average of all RMSE of the leads can be seen in Fig 8. The majority of the leads show a similar pattern in RMSE, with the exception of Lead6 and Lead9. (TIF) [file pone.0270559.s003.tif]

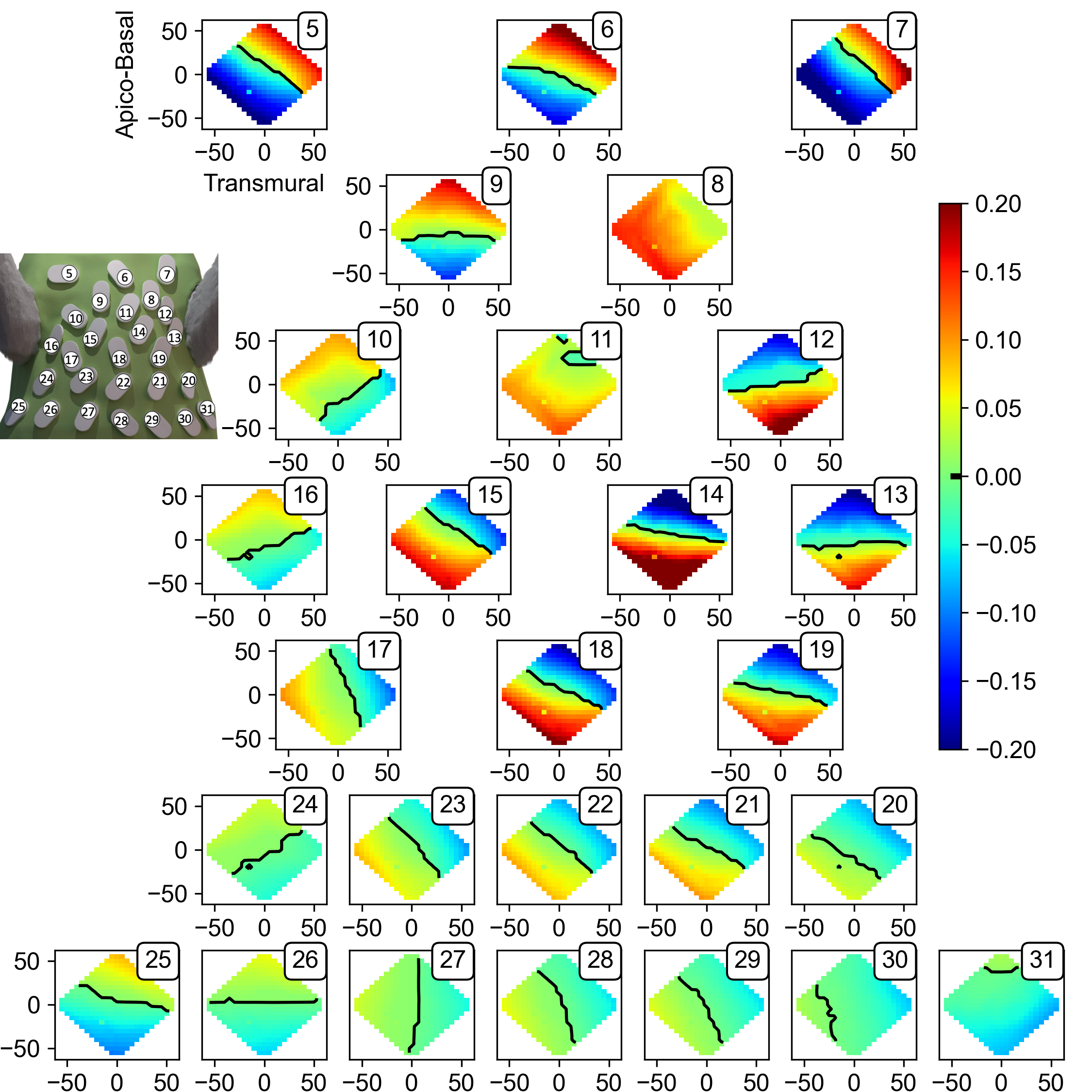

Supplement: S3 Fig — Maximum occurring amplitude during the T-Wave window, thus also showing the deflection direction of the T-Wave for the respective combinations of APD heterogeneity. The majority of leads show a dependency on both the transmural as well as the apico-basal gradient, with a few exceptions. Leads toward the lower middle of the torso are solely influence by the transmural gradient and Lead9,Lead12,Lead13, Lead14 solely by the apico-basal gradient. (TIF) [file pone.0270559.s004.tif]
